# Effects of Cyclin Dependent Kinase 9 inhibition on zebrafish larvae

**DOI:** 10.1080/15384101.2016.1231283

**Published:** 2016-10-07

**Authors:** Gianfranco Matrone, John J. Mullins, Carl S. Tucker, Martin A. Denvir

**Affiliations:** a British Heart Foundation Centre for Cardiovascular Science, The Queen's Medical Research Institute, The University of Edinburgh, Edinburgh, UK; b Department of Cardiovascular Sciences, Methodist Hospital Research Institute, Houston, TX, USA

**Keywords:** CDK9, flavopiridol, morpholino, pharmacology, phenotype, zebrafish

## Abstract

CDK9 is a known regulator of cellular transcription, growth and proliferation. Small molecule inhibitors are currently being developed and assessed in clinical trials as anti-cancer drugs. The zebrafish embryo provides an ideal model to explore the effects of CDK9 inhibition in-vivo. This has not been adequately explored previously at the level of a whole organism. We have compared and contrasted the effects of pharmacological and molecular inhibition of CDK9 on somatic growth, apoptosis and cellular proliferation in zebrafish larvae between 0 to 120 hours post fertilisation (hpf) using flavopiridol, a selective CDK9 antagonist, and CDK9-targeting morpholino. We demonstrate that the inhibition of CDK9 diminishes cellular proliferation and increases apoptosis. Subsequently, it affects somatic growth and development of a number of key embryonic structures including the brain, heart, eye and blood vessels. For the first time, we have localized CDK9 at a subcellular level in whole-mounted larvae.

This works shows, at a high-throughput level, that CDK9 clearly plays a fundamental role in early cellular growth and proliferation.

## Introduction

Cyclin-dependent kinase (CDK)9 is a regulatory molecule activated following binding to Cyclin T,^1^ forming a heterodimer that is the core element of the positive-acting transcription elongation factor (P-TEFb).^2^ In keeping with its central role in transcription regulation,^3^ CDK9 has also been implicated in abnormal cellular responses linked to cancer and HIV.^4^ This has focused attention on development of new therapies targeting pathways linked to CDK9.

So far, several CDKs inhibitor compounds have been developed with a range of selectivity for CDK9: Flavopiridol,^5^ Roscovitine,^6^ iCDK9,^7^ DRB,^8^ SNS-032,^9^ RGB-286147^10^ and AT7515.^11^ Flavopiridol, Roscovitine and SNS-032 are the best known CDK9 inhibitors tested in clinical trials, particularly as anticancer agents.^12-15^ These compounds inhibit several CDKs: roscovitine mainly inhibits CDK2, 5, 7 and 9, SNS-032 inhibits CDK2 and CDK9 while flavopiridol has high selectivity for CDK9, Ki <3 nmol/L compared with Ki values of 40 to 70 nmol/L for cell-cycle CDKs.^16^

Flavopiridol is a synthetic flavone which is structurally related to a natural molecule derived from *Dysoxylum binectariferum*, an indigenous plant from India.^17^ It has been tested in-vitro in several cell models of pathology, including human chronic lymphocytic leukemia cells,^18^ glioblastoma cells,^5^ leucocytes^19^ and smooth muscle cells.^20^ While in-vitro assays, using cell culture and bioinformatics tools, can provide a reasonably thorough assessment of toxicity many novel drug candidates can display off-target effects in-vivo at the level of whole organism which may not be apparent from these types of analyses. There is therefore a need to assess off-target effects at the level of a whole organism in order to balance the potential therapeutic benefits and risks for ongoing costs of drug development and ultimately for the patient who might receive the drug at some later date.

The zebrafish, Danio rerio, has emerged as a pliable vertebrate model organism to study physiological, pharmacological and pathologic processes quickly and at relatively low cost. Larvae show a high degree of permeability to small molecules making them well suited for testing and screening drugs targeting complex biological processes.^21^ Single and multiple compounds can be readily assessed,^22^ during development within 1–5 d of fertilisation. Experimental readouts can include detailed structural assessment, growth rate, histological studies including immunohistology. Indeed, zebrafish-based drug screening assays are increasingly used as part of routine preclinical safety evaluations of novel pharmacological compounds due to their ability to accurately predict toxicity in mammals. In this work, we tested the CDK9 inhibitor flavopiridol in zebrafish larvae assessing its effects on survival, growth, in-vivo cell proliferation and apoptosis. We showed that CDK9 knockdown by CDK9-targeted morpholino injection mimics the pharmacological effects of flavopiridol on cell apoptosis and proliferation.

## Results

### Survival and phenotype: Effects of flavopiridol and CDK9 morpholino

**Figure 1.**
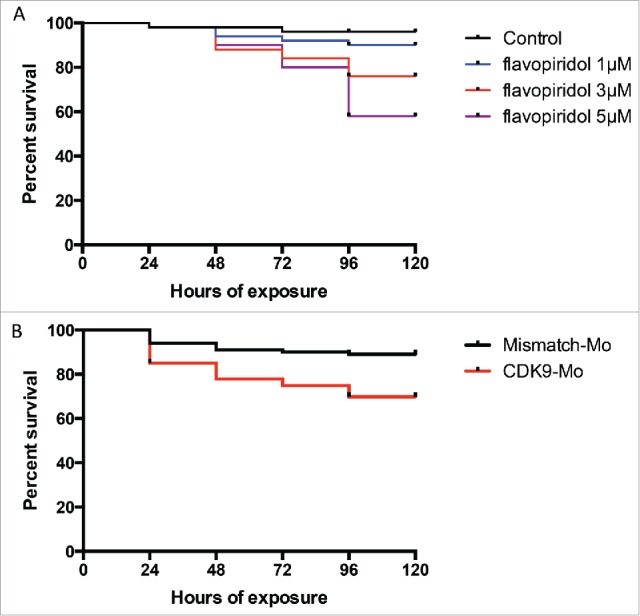
Kaplan-Meier survival curve following exposure to flavopiridol or morpholino injection. Survival rate in zebrafish embryos following continuous exposure to flavopiridol (at least n = 100 per group) in the range 1–5µM (A), from 24hpf up to 120 hpf or injection with morpholino 0.2ng/embryo (at least n = 100 per group) (B). Surviving embryos were counted every 24 hours until 120hpf.

**Figure 2.**
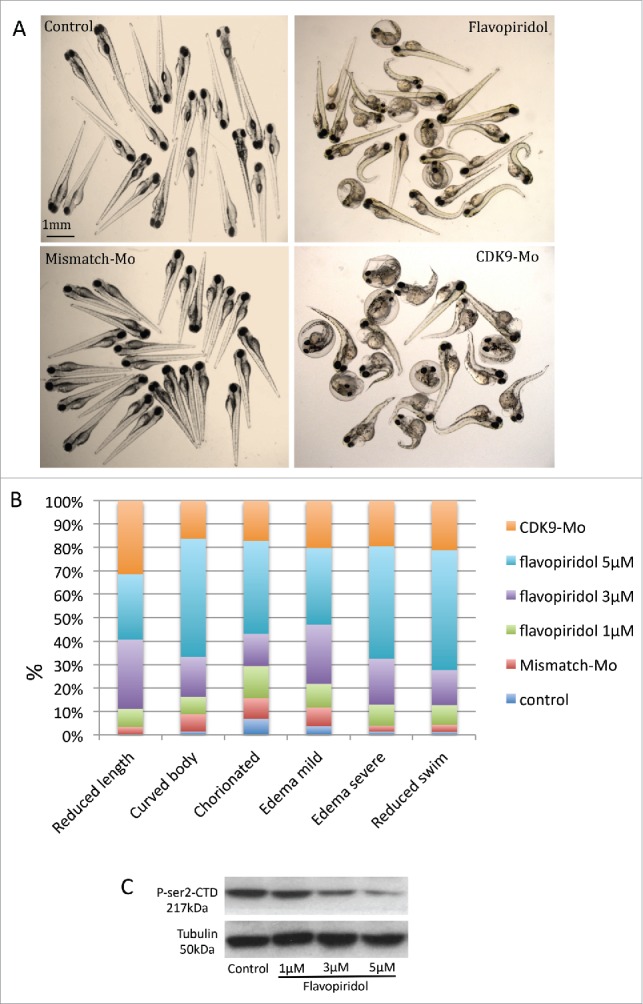
Analysis of zebrafish embryo phenotype following exposure to flavopiridol or morpholino injection. (A) Zebrafish embryos at 72 hpf continuously exposed to flavopiridol in the range 1–5µM from 24 hpf up to 120 hpf (at least n = 100 per group), or injected with morpholino 0.2ng/embryo (at least n = 100 per group). (B) Stacked column chart showing phenotypic traits observed following CDK9 inhibition. (C) Flavopiridol dose-dependent inhibition of CDK9 activity. Western blotting for Phospho Serine2 in the Carboxy-Terminal Domain (P-Ser2-CTD) of the RNA pol II. Serine 2 in this complex is phosphorylated by CDK9 when this is active. Tubulin was used as loading control.

Absence of swim activity, heart beat and tail blood flow were used as criteria to differentiate a viable from a non-viable larva. Kaplan-Meier curve showed that at 120 hours post-fertilisation (hpf), i.e. 96 hour post-exposure, the recorded survival was 92%, 75% and 57%, respectively in the group of larvae exposed to flavopiridol at 1 µM, 3 µM and 5µM (Fig. 1A). In comparison, CDK9 morpholino splice blocking injected embryo group showed 72% survival (Fig. 1B). Embryo phenotypic traits were analyzed at 72 hpf (Fig. 2). At the concentration of 5µM larvae malformations, such as curved body (50%) and edema (82% for both mild and severe) were commonly observed (Fig. 2A and B), whereas at the concentration of 3µM these malformation were less frequent, respectively 17% and 47%, although CDK9 activity was still reduced (Fig. 2C). At 5µM, 40% of embryos were still chorionated and 27% showed reduced total body length, whereas at 3µM these values were 12% and 30%, respectively, compared to control. A dose-dependent inhibition of CDK9 activity was observed at each of the 3 concentrations tested (1, 3 and 5 µM) as a progressive reduction in phosporylation of the target of CDK9, *i.e.*, serine 2 residue of the carboxy-terminal domain (P-Ser2-CTD) in the RNA pol II (Fig. 2C). On the basis of the findings in this dose response studies, flavopiridol 3µM was adopted thereafter in for all subsequent experiments. CDK9 morpholino injected embryos showed similar phenotypic traits as embryos treated with flavopiridol 3µM (Fig. 2B). Once again a range of concentrations of morpholino were tested and a final concentration was selected based on a balance of effective reduction in CDK9 levels, minimal phenotypic abnormalities in whole embryos and embryo survival of greater than 70% at 72hpf.

### Flavopiridol affects cell death and proliferation

**Figure 3.**
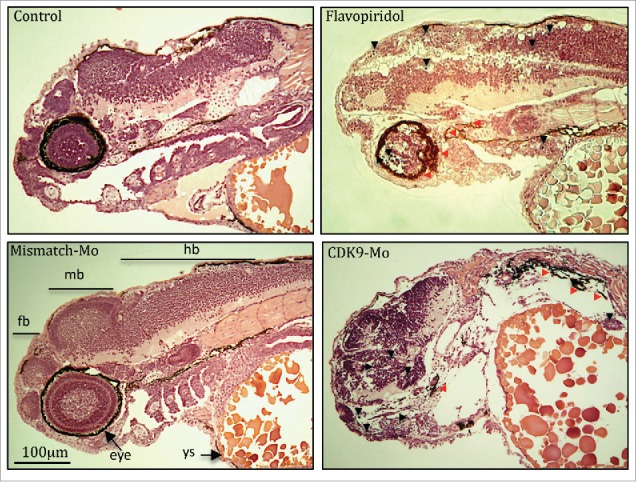
Haematoxylin and eosin histological staining. Zebrafish embryos (Wik, wild type strain) injected with CDK9-targeting morpholino or exposed to Flavopiridol 3uM showed increased appearance of apoptotic/necrotic bodies (black and red arrowheads, respectively), particularly in the brain area, with underdeveloped forebrain and midbrain, compared to control (black arrow). Fb, forebrain; mb, midbrain; hb, hindbrain; Ys, yolk sac.

**Figure 4.**
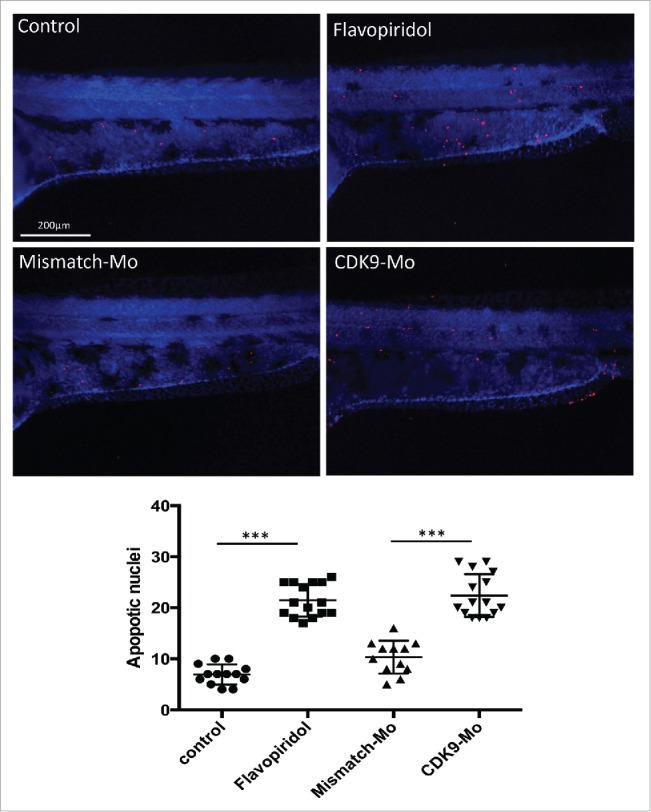
Effects of CDK9 inhibition on apoptotis. Lower panels – Terminal deoxynucleotidyl transferase dUTP nick end labeling (TUNEL) immunostaining. Zebrafish embryos (Wik, wild type strain) at 72 hpf injected with CDK9-targeting morpholino or continuouly exposed to Flavopiridol 3μM showed significant increase in the appearance of apoptotic bodies (number of TUNEL positive nuclei) compared to control, counted in the trunk region and reported in the scatter graph. At least n = 12 embryo per group; data were statistically analyzed by student t-test; *** = ≤0.001.

**Figure 5.**
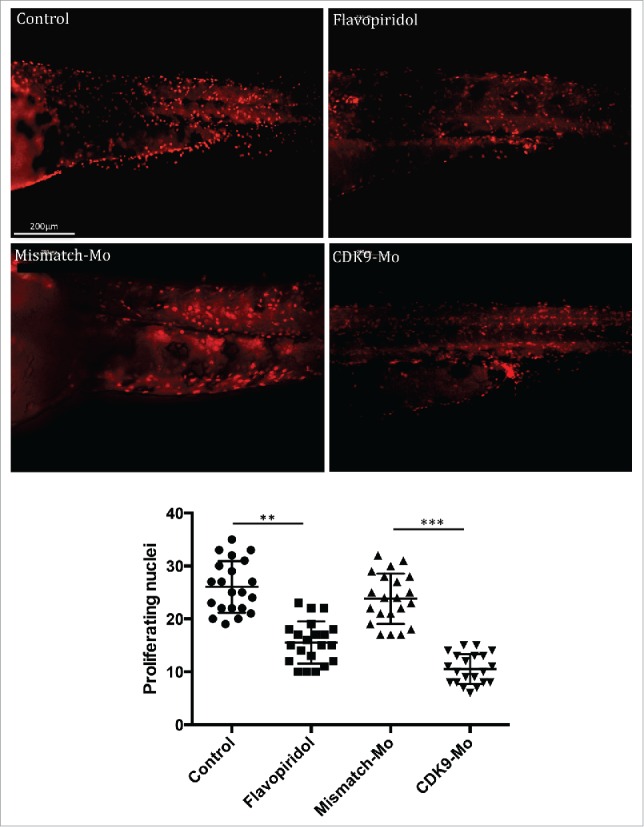
BrdU immunostaining. Injection of CDK9-targeting morpholino or exposure to Flavopiridol reduced significantly the number of BrdU positive nuclei compared to controls. Brdu figures were counted in the tail region as shown in these images and reported in the scatter graph. At least n = 20 embryo per group; data were statistically analyzed by student t-test; ** = ≤0.01, *** = ≤0.001.

H&E analysis showed underdeveloped forebrain and midbrain in embryos exposed to flavopiridol and features consistent with increased apoptosis compared to controls (Fig. 3, black arrowheads). Defects in eye development were also observed, in particular small eye with general or localized necrosis (Fig. 3, red arrowheads). TUNEL assay showed a significant increase in apoptotic nuclei in larvae exposed to flavopiridol compared to controls (Fig. 4). In contrast, BrdU immunostaining showed a significant reduction in the number of dividing cells compared to controls (Fig. 5). In both TUNEL and BrdU assays, CDK9-targeted morpholino treatment of larvae showed very similar results as for flavopiridol.

### In situ whole embryo CDK9 immunohistochemistry

**Figure 6.**
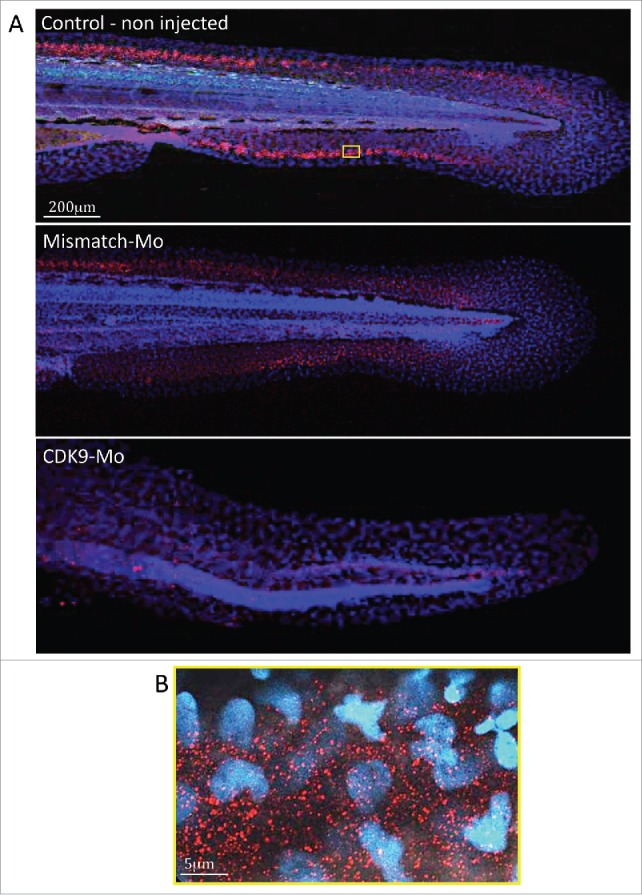
Immunostaining for CDK9 in whole larvae. (A) Confocal images of zebrafish embryo (Wik, wild type) 72 hpf control non-injected (above), injected with mismatch morpholino (middle) or CDK9-targeted morpholino (low) and immunostained with anti-CDK9 antibody, in red, and counterstained with DAPI. The staining shows diffuse presence of CDK9, in both nucleus and cytoplasm. The small yellow boxed area in a control non injected larva is shown at higher magnification in (B).

Immunostaining in whole-mount larvae identified ubiquitous presence of CDK9 throughout the embryo that was most apparent in the tail region (Fig. 6). Confocal imaging, at low magnification, showed a high intensity of CDK9 staining in a linear pattern around the tail region (Fig. 6A and B), probably coinciding with the growth plate of the tailfin. At high power magnification (Fig. 6C) there was a more punctate pattern of staining with distribution predominantly in the cytoplasm with less frequent staining in the nucleus. This pattern was not observed in larvae injected with CDK9-targeted morpholino where staining was significantly reduced confirming successful knockdown of CDK9 protein (Fig. 6C).

## Discussion

CDK9 inhibition is currently being evaluated in pre-clinical and clinical studies for the treatment of a number of different cancers mainly in combination with other chemotherapeutic agents.^3,6^ Flavopiridol acts by competing with ATP at the ATP-binding site of CDK9 and has been shown to decrease global levels of transcription in Drosophila,^23^ in HeLa or 293 cells^24^ and in chronic lymphocytic leukemia cells.^23^ This global inhibition of transcription is similar to that seen with other compounds known to target transcription such as Actinomycin D^25^ and DRB.^26^

We have shown that flavopiridol causes CDK9 inactivation as confirmed by Western blot analysis showing reduced phosphorylation of the Serine 2 (P-Ser2), the direct target of CDK9.^27^ Chao and Price^16^ found a similar result in Drosophila where they detected reduced^32^ P-incorporated-RNA polymerase II following exposure to flavopiridol. Decreased P-Ser2 and reduced transcription could explain the embryonic developmental delay observed in this work as shown by a reduction in total body length and a high proportion of chorionated embryos at 72 hpf. CDK9 activity, but not gene and protein expression, has been shown to increase in mouse myocardium via Gq, calcineurin and chronic mechanical signals for hypertrophic growth).^28^ CDK9 has also been shown to decrease as neutrophils age in culture and enter apoptosis.^29^ Indeed, we found an increased apoptosis (TUNEL staining), more evident in the brain structures and eye, and suppressed cell proliferation (BrdU staining) in the zebrafish whole embryo following CDK9 inhibition that could well explain the developmental defects observed in-vivo (Fig. 2A & B) and in H&E histology (Fig. 3). We have also shown that flavopiridol appears to cause a paradoxical increase in the levels of CDK9 protein and cardiac-related genes.^30^ This raises the prospect that a hitherto undefined feedback mechanism induced by flavopiridol exposure could be responsible for this finding. Indeed, Garriga et al.^25^ reported that despite the impact on general transcription, a significant number of transcripts are rapidly down- or upregulated following treatment of cultured human glioblastoma cells with flavopiridol. Moreover, specific inhibition of CDK9 activity using a dominant negative form of CDK9 leads to a distinctive change in the pattern of gene expression compared to that obtained with flavopiridol.^25^

There are several possible explanations for these observations. Flavopiridol may alter transcription by other mechanisms in addition to CDK9 inhibition or may inhibit other CDKs, although with lower efficacy. Indeed, the phosphorylation of Serine 5 on the CTD of the RNAPII is reduced by flavopiridol via its inhibitory effect on CDK7 in human glioblastoma cells.^5^ Furthermore, a recent screen seeking binding inhibitors to a panel of 119 kinases indicated that flavopiridol also binds the transcription regulator Calcium/Calmodulin kinase 1 with higher affinity than other CDKs and CDK-related kinases.^31^ CDK9 was unfortunately not specifically tested or reported in this study.

Flavopiridol has been shown to have potent antiproliferative effects on 60 human cancer cell lines in the US National Cancer Institute screen panel^32^ and is currently being evaluated in numerous studies for treating haematological and solid cancers. In the cardiovascular system, flavopiridol is known to inhibit smooth muscle cell proliferation and migration in vitro. We showed that flavopiridol affects cardiac development, performance and cardiomyocyte proliferation in the zebrafish.^30^ Considering these well characterized downstream effects of flavopiridol its effects on the heart are not unexpected.

Indeed, there appears to be a direct cardiotoxic effect of CDK inhibitors, as clinical use of these molecules have shown.^29^ However, the mechanism by which they exert their cardiotoxicity is not well understood. Hasinoff and Patel^33^ showed in neonatal rat ventricular cardiomyocytes that flavopiridol, and other kinases inhibitors, induce the release of the cytosolic enzyme lactate dehydrogenase into the media, a widely used measure of drug-induced damage to cardiomyocytes.^33^ An additional cardiotoxic mechanism might involve increased apoptosis of cardiomyocytes since flavopiridol is well recognized to induce programmed cell death in certain settings.^34^

### CDK9 immunostaining

CDK9 immunostaining in the whole embryo suggests widespread distribution of CDK9 throughout the body with more intense staining along the length of the tailfin. This ubiquitous presence supports the hypothesis that CDK9 represents a master regulator essential in early development.^35^ Higher magnification revealed a punctate distribution predominantly in the cytoplasm but also in the nucleus. Conversely, Dow et al.^36^ immunostained HeLa cells with the phospho-Thr186-CDK9 antiserum, showing the activated form of CDK9, and reported as a predominantly nucleoplasmic localization of CDK9 staining. Other reports support a predominantly nuclear localization of CDK9.^35,37^ In our experiments we used a CDK9 antiserum that labels either the phosphorylated or the non- phosphorylated forms of CDK9. Unfortunately, a phospho-Thr-CDK9 antibody for zebrafish was not available and therefore we could not distinguish between the 2 isoforms. However, Napolitano et al.^38^ indicated that CDK9 was predominantly located in the nucleus although there was also evidence of cytoplasm staining. These authors suggested that CDK9 is actively exported from the nucleus to the cytoplasm and that leptomycin B, a specific inhibitor of nuclear export, inhibits this process.

A further interesting finding in our immunostaining experiments is that the CDK9 cytoplasmic punctae appeared to be of different sizes. This is consistent with previous work suggesting that CDK9 exists in complexes of different size. One study found a 1:1:1 ratio for CDK9, cyclin T1 and HEXIM molecules in the large P-TEFb complex.^39^ However, since HEXIM homodimerizes through its coiled-coil regions, it was proposed that 2 HEXIM molecules and one 7SK snRNA associate with 2 cyclin T1/CDK9 heterodimers form the inactive P-TEFb complex.^40,41^

### Limitations of flavopiridol as a therapeutic drug

Flavopiridol has been proposed as treatment for several conditions due to its selectivity for CDK9 over other CDKs. However, flavopiridol is not selective for any particular organ or cell type and hence could have widespread and non-specific toxic effects on highly proliferative tissues such as the liver or bone marrow. There is therefore scope to develop tissue or cell-specific anti-CDK9 drugs. This possibility arises due to the presence of 2 different CDK9 isoforms, CDK9^55^ and CDK9.^42^ These are expressed differentially in certain cell types and a first step toward finding a more cell-specific compound would be a careful assessment of CDK9 isoform expression in different tissue types.

## Material and methods

### Ethical approval

All experiments were approved by the local ethics committee and conducted in accordance with the United Kingdom Animals (Scientific Procedures) Act 1986 in an approved establishment.

### Zebrafish maintenance

Zebrafish husbandry, embryo collection and maintenance were performed according to accepted standard operating procedure.^42^ The Wik (wild type) strain was used for all experiments and staged according to Kimmel.^43^ Larvae were maintained at 28.5°C on a 14 h light/10 h dark cycle in egg water until dechorionated and then in embryo medium.^44^ Larvae were anesthetized in a solution of Tricaine 20 µmol/L (µM) (ethyl 3-aminobenzoate methanesulfonate, Sigma, cat. E10521) and euthanised with an overdose of the same compound. All experimental procedures were performed at room temperature (RT, 23°C).

### Pharmacological treatment of larvae

Zebrafish larvae (24 hpf) were placed in embryo medium containing flavopiridol (Sigma, cat. F3055) 3 µmol/L (µM) diluted in 1% DMSO carrier solvent. Solutions were replaced at 48, 72 and 96 hpf. Control larvae were exposed at DMSO 1%. The drug concentration of 3 µmol/L was selected after a series of experiments assessing the concentration of flavopiridol which resulted in minimum toxicity to the whole embryo while also resulting in a significant decrease in CDK9 activity confirmed by reduced phosphorylation of the serine 2 residue of RNA polymerase II.^45^

Initially, exposure to the Flavopiridol was started just after fertilization but this caused death of all larvae by 24 hpf. Exposure to flavopiridol was therefore started at 24 hpf when survival was found to be considerable increased. Survival data for embryos treated from 24 to 96 hours are plotted on a Kaplan-Meier curve (Fig. 1).

### Morpholino injections

A 0.5 nanolitre solution containing 0.2ng of CDK9-targeting morpholino (Mo) (Gene Tools) was injected in one to 2-cell stage larvae just beneath the blastoderm using a pulled glass pipette using a standard injector (Narishige, Microinjector IM300). Successful injection was assessed under fluorescence microscope by the red tag lissamine at the 3′end of the Mo. Two different morpholinos were used. The translation blocking Mo that binds the CDK9 mRNA translation initiation complex including the ATG triplet. This Mo (5′- CTTCCGGTTTTGTCGCGCTGCATCC -3′, (NC_007132.6)) resulted in a high mortality of around 50% of embryos by 24 hpf even at very low concentrations. Whereas injection of the CDK9-Mo splice blocking, designed against exon 3 and intron 3 (5′- GGTGCATTTTCTTACCCCTTCTTTC -3′, (NM_212591.1)) resulted in a better survival at 24 hours despite effective knockdown of CDK9 protein and hence this was used for all subsequent experiments. A mismatch Mo was used as a control (5′- GGTcCATTTTgTTAgCCgTTgTTTC -3′).

### BrdU assay

BrdU (5-Bromo-2′-deoxyuridine) labeling was performed as described in Laguerre et al.^46^ and modified as follows. Live larvae were placed in a 90mm diameter petri dish and incubated in BrdU (Sigma-Aldrich, cat. B5002) 10 mM in embryo medium (EM) with 15% DMSO (Sigma, cat. D2650) for 20 min, on a surface of ice; thermal shock allows BrdU penetration within the embryo. The petri dish was then removed from the ice and larvae rinsed twice with fresh embryo medium at 28.5°C before recovering in the incubator at 28.5°C for 2 hour. Larvae were then euthanised in tricaine and fixed overnight in PFA 4% and rinsed 3 times in PBS containing Triton-X100 0.1% (PBS-Tx100).

To aid antibody penetration, larvae were digested with Proteinase K (Sigma, cat. P2308) 10μM for 20min at RT and then rinsed several times in PBS-Tx100. They were then re-fixed for 30min in PFA 4% before being rinsed twice in PBS and then twice in HCl 2N before being incubated at RT for a further 1 hour in HCl 2N. They were rinsed 3 times in PBS and incubated for 1–2 hours in bovine serum albumin (BSA) (Sigma, cat. A7906) 3% blocking solution at RT or overnight (ON) at 4°C while gently shaking. Larvae were then incubated for 2h in anti-BrdU (1:100 rat; Dako, cat. M0744) at RT or ON at 4°C. After rinsing several times, larvae were incubated for 2h with anti-mouse TRITC (1:500) in the dark. Larvae were then mounted on chambered slides (Microscope cavity slides 2 cell, Hawksley, cat. 2CS000) with ProLong→ Gold antifade reagent (Life Technologies, cat. P36930), examined on a fluorescence microscope Zeiss Axioskop II MOT Plus (Carl Zeiss) using a 40x objective and digital images captured for later analysis. BrdU positive nuclei were counted in at least 5 larvae per group (Fig. 4).

The sampling strategy employed for image capture involved randomly selecting 2 fields from embryo body length. Images were downloaded to a computer for image analysis with ImageJ software. For each image collected, a region of interest of 10,000 µm^2^ was used within the image to count BrdU positive nuclei (Fig. 5).

### CDK9 immunostaining

Larvae were euthanized in Tricaine 1mM and fixed in 4% paraformaldehyde (PFA, Sigma, P6148). Then, larvae were permeabilized in proteinase K (10 µg/ml), for 20 min at RT, then washed in PBS-Tx100 (0.1%) and blocked in Bovine Serum Albumin 5% in PBS for 3 h. Larvae were incubated with anti-CDK9 antibody (Cell Signaling Technology, C12F7, rabbit 1:100 in PBS), followed by incubation with anti-rabbit antibody (Alexa fluor, Dako, 1:500). Subsequently, larvae were incubated in DAPI (1:1000, Sigma, cat. D9542) for 15 minutes, washed in PBS and then mounted in glycerol 100%. Confocal microscopy (Leica SP5) was used to capture z-stack images of whole larvae.

### Histopathology

Haematoxylin & Eosin (H&E) staining was used to examine histological features of cells and organs.^47^ After fixation in 4% PFA for 3h at RT or ON at 4°C, specimens were dehydrated through an ascending ethanol series (from 25% – 100% in 5 steps) and embedded in paraffin in a transverse or sagittal orientation. Serial 5μm tissue sections were cut on a microtome and stained with H&E, according to standard protocols.^47^ H&E is a general histological stain for cell nuclei (haematoxylin, colored blue); other structures, i.e. cytoplasm, collagen and muscle fibers will be stained with eosin and therefore be colored red. Sections were observed under compound microscope and images captured using a standard color camera. A qualitative analysis of typical cytomorphological alterations of apoptotic cells (cell shrinkage, eosinophilic dense cytoplasm, pyknotic nuclei, karyorrhexis) was performed.

### Whole-mount TUNEL assay

The TUNEL method is used to assay the endonuclease cleavage products by enzymatically end-labeling the DNA strand breaks.^48^ Apoptotic cell death in whole-mount zebrafish was detected according to a modification of the ApopTag rhodamine In Situ Apoptosis Detection kit (Chemicon, cat. S7165) protocol. Larvae were fixed in 4% paraformaldehyde (PFA) at 4°C, washed in PBS, permeabilized with proteinase K (10µg/ml) followed by 2 further washes in PBS. They were then fixed again in 4% PFA, placed in prechilled ethanol:acetic acid (2:1) at −20°C for 10 min, then washed in PBS-T (PBS 1X, 0.1% Tween-20) 3 times before incubation in equilibration buffer and further steps as recommended by the manufacturer. TUNEL assay staining was quantified by counting positive staining puncta in the whole embryo from z-stack confocal images using ImageJ.

### Analysis of the phenotype

Whole embryo phenotype following treatments were described at 72 hpf on the basis of morphologic and functional characteristics under bright field microscopy and reported graphically (Fig. 2) as a stacked column graph. In each column the percentages of the defined phenotype obtained in each treatment were reported. Chorion phenotype represents embryos that are still located in their chorion at 72 hpf. Edema phenotypes refer to the severity of edema that surrounds the anteroventral part of the fish close to the heart. Curved body phenotype refers to larvae with an abnormal curvature in the longitudinal axis. Reduced body length phenotype assessed the percentage of embryos with body length, measured as the distance from the snout to the posterior tip of the notochord, less than the 10% of the average in the control group. Reduced swim phenotype refers to the percentage of embryo with low swim or absent swim standing at the bottom of the petri dish. Phenotype characterization was undertaken independently within our laboratory for each of the 2 CDK9 manipulations. At least 4 different clutches of larvae were assessed under each of the treatment groups.

### Statistical analysis

Experiments were performed in triplicate with on average 30–50 larvae per experiment, unless otherwise stated. Data are presented as mean ± standard error of the mean (SEM). Statistical analyses were performed using GraphPad Prism 5. For normally distributed data, the Student t-test was used to compare means between groups and Mann Whitney U test was used for non-normally distributed data. P values <0.05 were considered significant.

## Abbreviations

BrdU: 5-Bromo-2′-deoxyuridine
CDK9: cyclin-dependent kinase 9
Fb: forebrain; mb – midbrain
hb: hindbrain
Mo: morpholino
P-TEFb: positive-acting transcription elongation factor
TUNEL: Terminal deoxynucleotidyl transferase dUTP nick end labeling
Ys: yolk sac
